# Evaluating an anti-idiotype derived germline-targeting immunogen designed to elicit VRC01-class precursors in human antibody transgenic mice

**DOI:** 10.1128/msphere.00317-26

**Published:** 2026-09-01

**Authors:** Crystal B. Chhan, Yu-Hsin Wan, Andrew Wilcox-King, Leah J. Homad, Nicholas T. Aldridge, Roman Iureniev, Leonidas Stamatatos, Andrew T. McGuire

**Affiliations:** 1 Vaccine and Infectious Disease Division, Fred Hutchinson Cancer Center 561181, Seattle, Washington, USA; 2 Department of Global Health, University of Washington 150331, Seattle, Washington, USA; 3 Department of Laboratory Medicine and Pathology, University of Washington 189576, Seattle, Washington, USA; Capital Medical University Beijing Ditan Hospital, Beijing, China

**Keywords:** HIV-1, antibodies, transgenic mice, germline targeting, anti-idiotype

## Abstract

An effective HIV-1 vaccine will likely need to elicit broadly neutralizing antibodies (bNAbs) that bind relatively conserved regions of the otherwise highly variable envelope glycoprotein. Among these, VRC01-class bNAbs are a reproducible antibody class that bind the CD4-binding site through genetic features encoded by the VH1-2 heavy chain and a light chain containing a rare five-amino-acid-long complementarity-determining region 3 (CDRL3). We previously developed a germline-targeting immunogen, iv4/iv9, derived from anti-idiotypic monoclonal antibodies (ai-mAbs) that target these genetic signatures of VRC01-class bNAbs. Here, we applied structure-guided modification of iv4/iv9 that improved selective binding to VRC01 precursors *in vitro* and carried out immunizations in ATX-GK mice. These mice are transgenic for human antibody genes, producing a diverse, genetically human antibody repertoire. We found that ATX-GK mice harbor VRC01 precursors at frequencies lower than those found in humans. Class-switched VRC01 precursors were detected in a minority of mice immunized with a modified iv4/iv9 immunogen. Collectively, these results indicate that the ATX-GK mice have utility to evaluate VRC01-class germline-targeting immunogens but are a stringent model due to a low frequency of VRC01-class B cells. They further suggest that the ai-mAb immunogens evaluated herein will require further optimization to reproducibly prime VRC01-class B cells in ATX-GK mice.

**IMPORTANCE:**

An effective HIV-1 vaccine will likely need to elicit broadly neutralizing antibodies (bNAbs) that bind relatively conserved regions of the otherwise highly variable envelope glycoprotein. Among these, VRC01-class bNAbs are a reproducible antibody class that bind the CD4-binding site through genetic features encoded by the VH1-2 heavy chain and a light chain containing a rare five-amino-acid-long complementarity-determining region 3 (CDRL3). We previously developed a germline-targeting immunogen, iv4/iv9, derived from anti-idiotypic monoclonal antibodies that target these genetic signatures of VRC01-class bNAbs. Here, we evaluate the B-cell response to immunization with iv4/iv9 and with a structure-guided modified iv4/iv9 in ATX-GK mice. These modifications are intended to improve the selectivity of VRC01 precursors in a diverse polyclonal B cell repertoire. These mice are transgenic for human antibody genes, producing a diverse, genetically human antibody repertoire. We found that ATX-GK mice harbor VRC01 precursors at frequencies lower than those found in humans. Class-switched VRC01 precursors were detected in a minority of animals immunized with the modified iv4/iv9. Collectively, these results indicate that although the iv4/iv9 immunogen did not reproducibly elicit VRC01-class B cells, ATX-GK mice have utility to evaluate VRC01-class germline-targeting immunogens but are a stringent model due to a low frequency of VRC01-class B cells.

## INTRODUCTION

Human immunodeficiency virus-1 (HIV) is the causative agent of acquired immunodeficiency syndrome (AIDS) (1). HIV infects CD4^+^ T cells through the attachment and fusogenic activity of the envelope glycoprotein (Env), the sole virally encoded protein on the viral surface. The isolation and characterization of monoclonal antibodies that bind conserved regions of HIV Env and potently neutralize diverse viral isolates define critical sites of vulnerability that are relevant to vaccine design (2–4). Among these, VRC01-class antibodies target the CD4-binding site (CD4bs) and are among the most broad and potent of all broadly neutralizing antibodies (bNAbs) (5–14). Passive transfer or genetic expression of VRC01-class bNAbs protects animals from experimental HIV-1 infection (15–19). Additionally, human clinical trials showed that passive delivery of VRC01 reduced the incidence of infection with sensitive isolates (20, 21).

VRC01-class antibodies have been isolated from multiple HIV-infected donors (5, 6, 8–11). Despite having up to 50% sequence divergence, they bind conserved regions of the CD4bs in a similar manner (8–11, 22–24). Unique genetic features of VRC01-class antibodies, namely, a heavy chain (HC) derived from the VH1-2 gene paired with a light chain (LC) harboring a five-amino-acid-long complementarity-determining region 3 (5aa CDRL3), are key to this conserved mode of Env recognition (6, 12, 13, 22–25).

VRC01-class antibodies are highly somatically mutated (2, 3, 6, 9). However, unmutated VRC01 B-cell receptors (VRC01 BCRs) fail to recognize recombinant Env, which spurred efforts to develop germline-targeting immunogens to selectively target VRC01 B cell precursors and begin the process of maturation toward broad neutralization (26–33). Our group previously designed a bispecific molecule derived from anti-idiotypic monoclonal antibodies (ai-mAbs) called iv4/iv9 that can simultaneously target two genetic signatures found in all VRC01-class bNAbs (34). The iv9 arm targets the VH1-2 heavy chain, while the iv4 arm targets light chains harboring rare 5aa CDRL3. iv4/iv9 could identify genetically diverse VRC01 class precursor B cells among pools of naive human PBMCs, and selectively engage and activate inferred germline VRC01 (iGL-VRC01) B cells *ex vivo* and *in vivo* using a murine adoptive transfer system (34–36). A limitation of this model is that all the VRC01-class precursors have a fixed BCR, while putative VRC01 precursors in humans have diverse CDRH3s and can use multiple light-chain genes with 5aa CDRL3s (35, 37, 38).

Here, we investigated whether iv4/iv9 could selectively prime VRC01-class precursor B cells in a transgenic mouse model, ATX-GK from Alloy Therapeutics, which expresses a diverse, rearranging, genetically human antibody repertoire (39). Analysis of paired BCRs from ATX-GK mice indicates that VRC01-class precursor B cells are present at ~11-fold lower frequencies than they are in humans. Immunization with iv4/iv9 preferentially expanded B cells with VH1-2 heavy chains but did not select for VRC01-class B cells. The introduction of structure-guided targeted mutations into the iv9 arm lowered its affinity for VH1-2 heavy chains. Using B-cell sorting of pooled samples, we detected VRC01 precursor B cells with evidence of clonal expansion in an initial cohort of three mice immunized with the modified iv4/iv9, but not in a second cohort of three individually analyzed iv4/iv9mod-immunized mice. From these results, we conclude that while the ATX-GK mice can generate putative precursor VRC01 B cells, their frequency may be too rare to be reliably targeted by immunization with iv4/iv9.

## RESULTS

### Assessment of VRC01-class precursor frequency in ATX-GK mice

**Fig 1:**
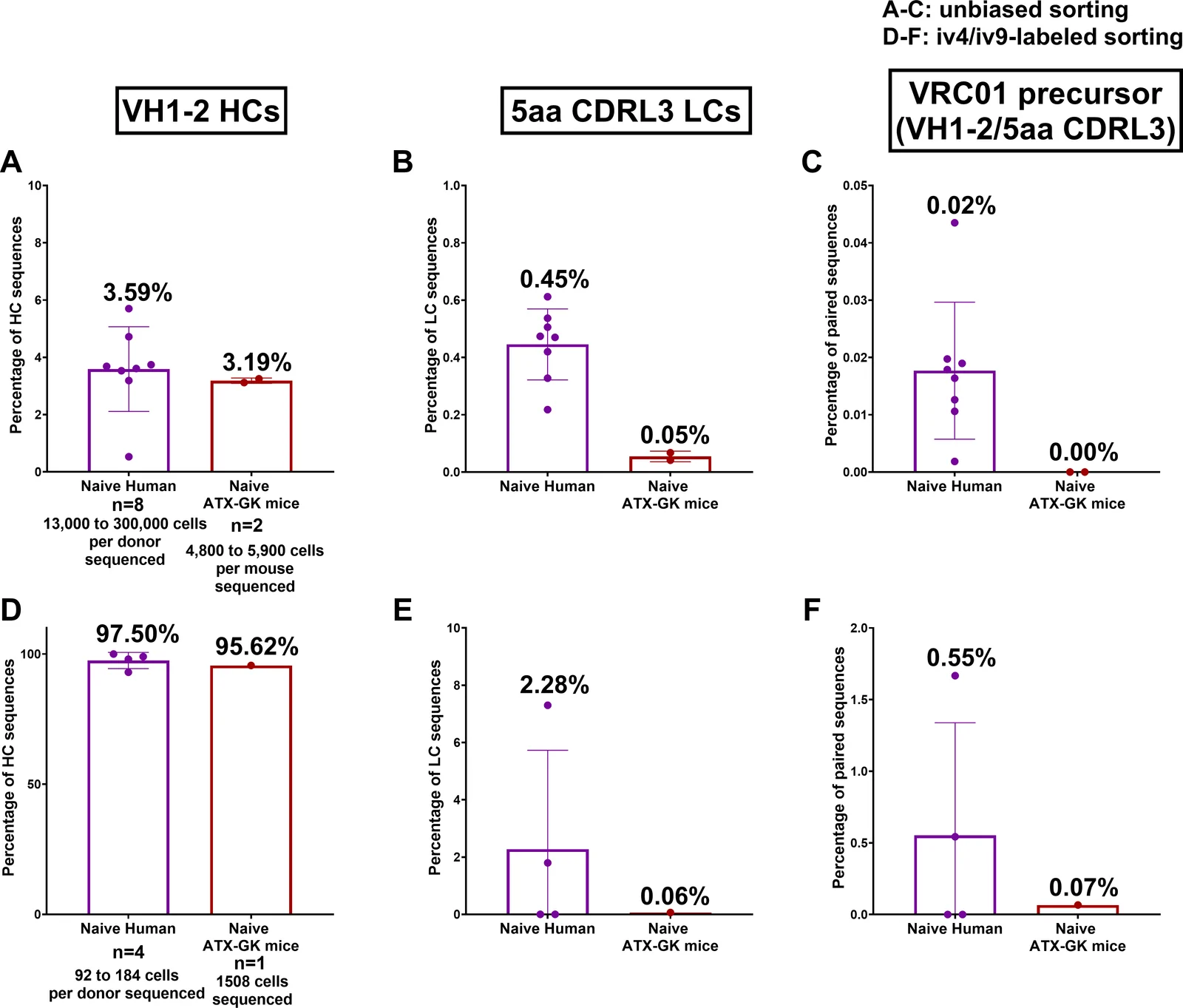
Frequency of BCRs with genetic characteristics of VRC01 class antibodies in naive ATX-GK mice. (**A–C**) Percentage of VH1-2 heavy chains (**A**), light chains with 5aa CDRL3 (**B**), and VRC01 precursors (**C**) observed among paired BCR sequences from circulating naive human B cells and from naive splenic B cells from ATX-GK mice identified by unbiased, high-throughput paired sequencing (39–41). (**D–F**) Frequencies of observed VH1-2 heavy chains (**D**), light chains with 5aa CDRL3 (**E**), and VRC01 precursors (**F**) among BCRs from ATX-GK splenic B cells sorted using iv4/iv9 as bait and subjected to high-throughput paired BCR sequencing. The frequencies in panels **D–F** are compared to those previously observed in naive human B cells sorted with iv4/iv9 (34). Bars and listed numbers represent the mean. Error bars represent the standard deviation.

Through unbiased, single-cell deep-sequencing of splenocytes, we confirmed that ATX-GK mice express a diverse, genetically human repertoire (39). We found that 3.19% of B cells in ATX-GK mice express VH1-2 heavy chains (HCs) (Fig. 1A), which is comparable to the frequency found in humans (40). Additionally, 0.05% of ATX-GK B cells express light chains with five-amino-acid-long third complementarity-determining regions (CDRL3), which is ~8-fold lower than in humans (39–41) (Fig. 1B). We were unable to identify a VRC01 precursor cell among 10,735 paired BCRs sequenced from two mice (Fig. 1C). Based on the observed frequencies of VH1-2 HCs and 5aa CDRL3 LCs, we estimate that putative VRC01 precursors (defined as B cells encoding both the VH1-2 heavy chain and 5aa CDRL3 light chain) are present at ~1 in 50,000 to 1 in 75,000 B cells in ATX-GK mice (Table S1). Assuming there are ~75 million B cells in a mouse, this translates to ~1,000–1,600 VRC01-class B cells per mouse (Table S1).

We previously demonstrated that antigen-specific sorting of B cells using fluorescently labeled iv4/iv9 as bait enriches for VRC01-class precursors in pools of naive human B cells (34). Here, we used this approach to sort splenic B cells from one naive ATX-GK mouse. Single-cell sequencing of iv4/iv9-sorted B cells showed a strong enrichment of B cells encoding VH1-2 HCs (95.62%), comparable to frequencies of iv4/iv9-sorted human B cells from peripheral blood in a previous study (34) (Fig. 1D). The frequency of B cells with 5aa CDRL3 LCs in ATX-GK mice was not enriched by iv4/iv9 sorting (0.06%) (Fig. 1E; Table S5). We identified one VRC01-class precursor, which we named PATXP1, among 1,508 sorted and sequenced B cells (Fig. 1F; Table S4). Taken together, we conclude that although the frequency of naive VRC01-class precursors is at least 11-fold lower in the ATX-GK mouse compared to humans, they can be identified by iv4/iv9 *ex vivo*.

### Optimization of a germline VRC01-targeting, anti-idiotypic, bispecific immunogen

**Fig 2:**
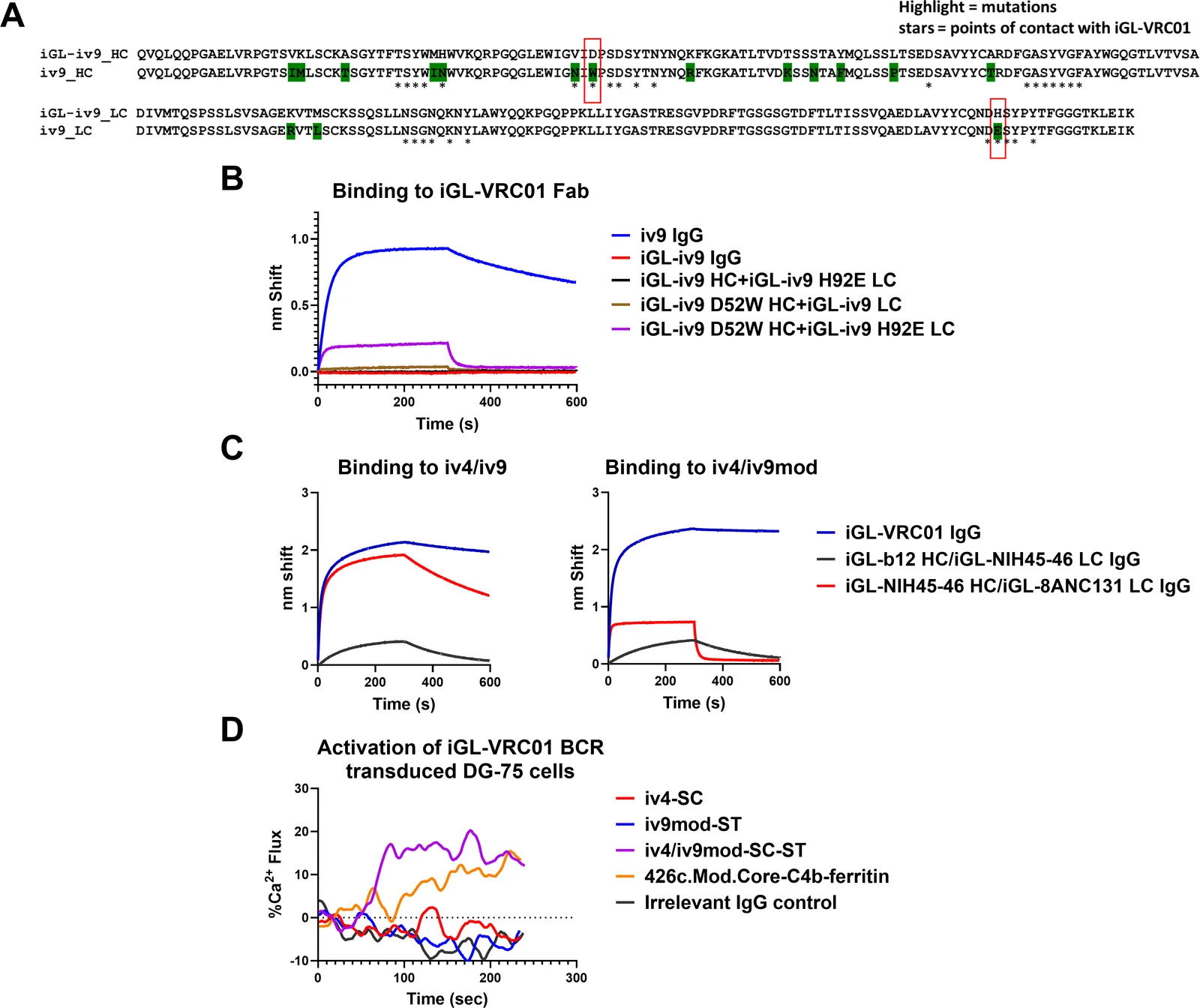
Structure-guided mutation of iv9 to reduce affinity for VH1-2 heavy chains. (**A**) Heavy-chain (HC) and light-chain (LC) sequences of iv9 aligned to the inferred germline sequences. Somatically mutated amino acids are highlighted in green, while residues that contact iGL-VRC01 in the co-crystal structure are denoted by an asterisk (34). Red boxes denote the Trp52_HC_ and Glu92_LC_ contact residues. (**B**) The indicated iv9 derivatives were tested for binding to iGL-VRC01 Fab using biolayer interferometry. (**C**) Binding of iv4/iv9 (left) and iv4/iv9mod (iGL-iv9 D52W H92E) (right) to iGL-VRC01 and the indicated chimeric mAbs was assessed using biolayer interferometry. (**D**) Measurement of intercellular calcium release in DG-75 cells stably expressing iGL-VRC01 BCRs in response to stimulation with 100 nM of iv4/iv9mod, iv4 Fab SC, iv9mod Fab ST, 426c.Mod.Core-ferritin, or anti-EBV AMMO1 IgG, as indicated (28, 42).

Our analysis indicates that 95.62% of iv4/iv9-sorted B cells expressed VH1-2 heavy chains, but only 0.07% of those iv4/iv9-sorted B cells were VRC01 precursors (Fig. 1D and F), which indicates that over 90% of B cells recognized by iv4/iv9 are off-target B cells expressing VH1-2 HCs with undesired light chains. The iv9 arm of the bispecific molecule has higher affinity for VH1-2 HCs than iv4 does for LCs with 5aa CDRL3s, primarily due to the faster off-rate of iv4 (34). We hypothesized that increasing the off-rate of iv9 would lower its affinity and make the bispecific molecule more selective for VRC01 precursors. We reasoned that if each arm has a fast-off rate, only avid engagement of VRC01 precursors would slow the off-rate of the bispecific molecule and improve selectivity for VRC01 precursors over cells with only VH1-2 heavy chains or 5aa CDRL3s. We first reverted all somatic mutations in iv9 to their inferred germline residues (iGL-iv9), which abrogated binding to iGL-VRC01 (Fig. 2A and B). Next, we selectively reintroduced residues D52W_HC_ and H92E_LC_ in iGL-iv9 which we predicted to be critical for iGL-VRC01 binding because they are (i) somatically mutated and (ii) bury substantial surface area on the VH1-2 heavy chain in the iGL-VRC01/iv9 complex (34) (Fig. 2A). Neither iGL-iv9 D52W_HC_ nor H92E_LC_ bound iGL-VRC01, while iGL-iv9 containing both D52W_HC_ and H92E_LC_ mutations conferred binding (Fig. 2B; Table S2). The binding of iGL-iv9 D52W_HC_ H92E_LC_ (herein called iv9mod) to iGL-VRC01 (*K*_*D*_ = 5.45 × 10^−6^ M) was weaker than the binding of iv9 (*K*_*D*_ = 6.05 × 10^−9^ M) with a faster off-rate (34) (Fig. 2B; Table S2). We next conjugated iv9mod to iv4 and tested binding to iGL-VRC01 or chimeric mAbs comprised of the iGL-NIH45-46 (a clonal variant of VRC01 isolated from the same donor) heavy chain paired with an irrelevant light chain or vice versa (8). Relative to iv4/iv9, iv4/iv9mod showed reduced binding to the iGL-NIH45-46 HC/iGL-8ANC131 LC chimera with a notably faster off-rate (Fig. 2C, red curves), while binding to the iGL-b12 HC/iGL-NIH45-46 LC chimera was unchanged (Fig. 2C, black curves). Under these experimental conditions, which permit avid binding, iv4/iv9 and iv4/iv9mod showed comparable binding to iGL-VRC01 (Fig. 2C, blue curves). We also verified that iv4/iv9mod could bind to naturally paired putative VRC01-class antibodies sorted from naïve human B cells using iv4/iv9 (PHVP6 and PHVP7) (34) or from unbiased high-throughput BCR sequencing (DK12) (40) (Fig. S1).

iv4/iv9mod and a germline-targeting Env (426c.Mod.Core-ferritin) both activated DG-75 cells engineered to express iGL-VRC01 BCRs using *in vitro* calcium flux assays, while the unconjugated iv4 and iv9mod Fabs could not (43) (Fig. 2D). These data indicate that, relative to iv4/iv9, iv4/iv9mod has reduced affinity for off-target B cells while retaining the ability to avidly engage and activate VRC01-class BCRs.

### VRC01 precursors detected in ATX-GK mice immunized with an anti-idiotypic immunogen

**Fig 3:**
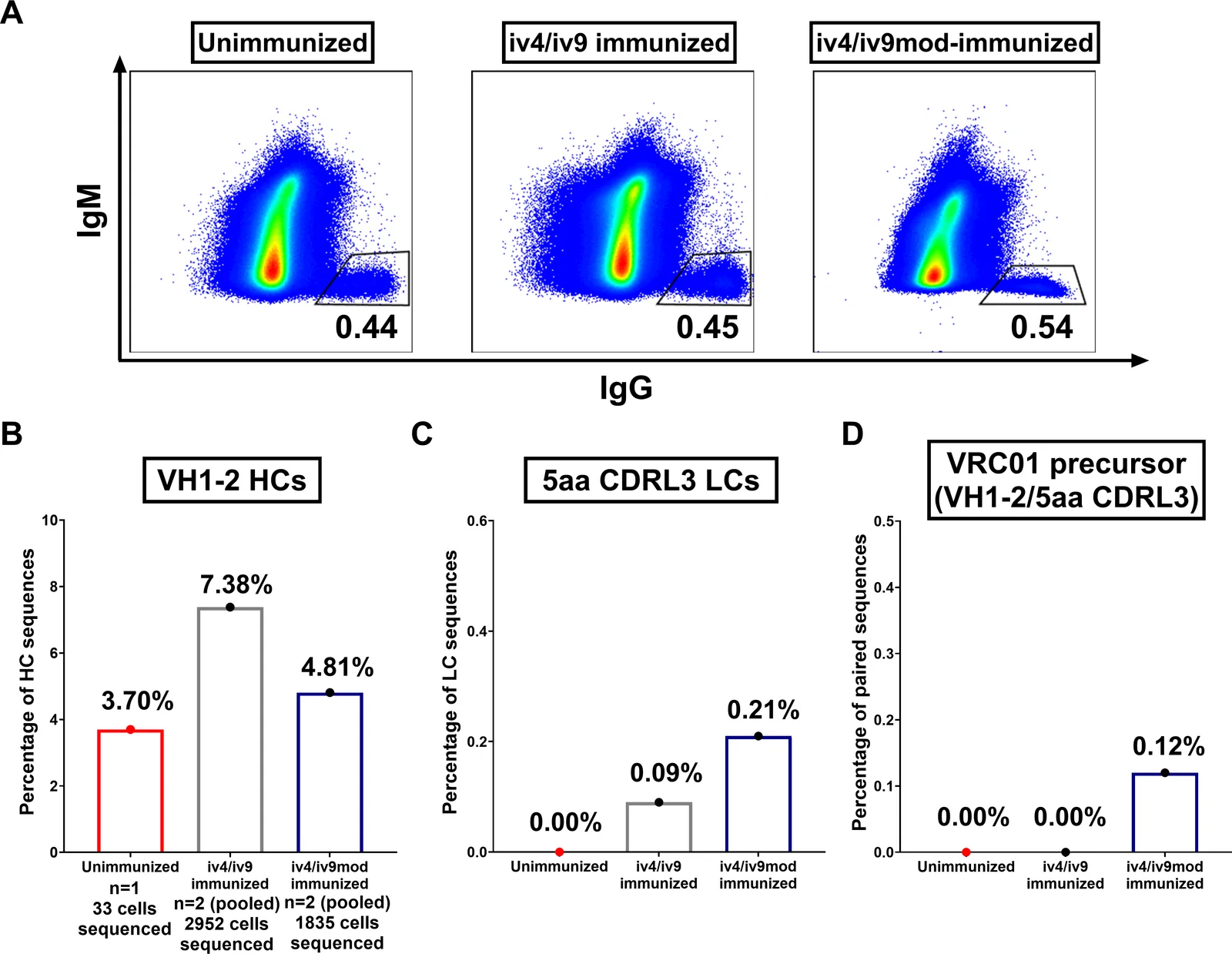
High-throughput sequencing of splenic IgG^+^ B cells from ATX-GK mice immunized with ai-mAbs. (**A**) Frequency of IgG^+^ cells of total splenic B cells from unimmunized, iv4/iv9-immunized, and iv4/iv9mod-immunized ATX-GK mice as indicated. (**B–D**) IgG^+^ splenic B cells were sorted from unimmunized (red bars), iv4/iv9-immunized (gray bars), or iv4/iv9mod-immunized ATX-GK mice (blue bars) and subjected to high-throughput paired, single-cell sequencing. Frequency of observed VH1-2 heavy chains (**B**), 5aa CDRL3 LCs (**C**), and VRC01-class BCRs (**D**).

To determine if iv4/iv9 and iv4/iv9mod could expand VRC01 precursors *in vivo,* ATX-GK mice were pre-primed with a peptide (2W1S) known to elicit a strong CD4^+^ T cell response in C57BL/6 mice (44). Then mice were immunized with iv4/iv9 (*n* = 2) or iv4/iv9mod (*n* = 3), both of which were tagged with 2W1S peptide and formulated with SMNP as an adjuvant (45). We had previously used this peptide fusion strategy to recruit pre-existing T cell help and improve B-cell responses elicited by an anti-idiotypic mAb (36, 46). We reasoned that a portion of vaccine-responding B cells would be class-switched. Fourteen days after immunization, we pooled at least 20 million splenic B cells from two mice per group, sorted IgG^+^ B cells, and subjected them to high-throughput paired sequencing (Tables S3 and S5). We included an unimmunized sample as a control. All three groups had comparable frequencies of IgG^+^ B cells (Fig. 3A; Table S5). From the recovered sequences, we found that 3.70% of IgG^+^ B cells in unimmunized mice expressed VH1-2 HCs (Fig. 3B), which was comparable to the frequency of VH1-2 HC-expressing B cells in the naive IgM repertoire (Fig. 1A). Mice immunized with iv4/iv9 (mice #7 and #8) had an elevated frequency of VH1-2 IgG^+^ B cells (7.38%), while mice immunized with iv4/iv9mod (mice #9 and #10) had an intermediate frequency (4.81%, Fig. 3B; Table S5). The frequency of LCs with 5aa CDRL3s was higher in the iv4/iv9mod group (0.21%) compared to the unimmunized mouse (0.00%) and the iv4/iv9-immunized mice (0.09%) (Fig. 3C). Among the IgG^+^ B cells from the iv4/iv9mod-immunized mice, we identified two VRC01-class precursors (PATXP2 and PATXP3), while none were observed among the iv4/iv9-immunized mice or unimmunized controls (Fig. 3D; Tables S4 and S5).

**Fig 4:**
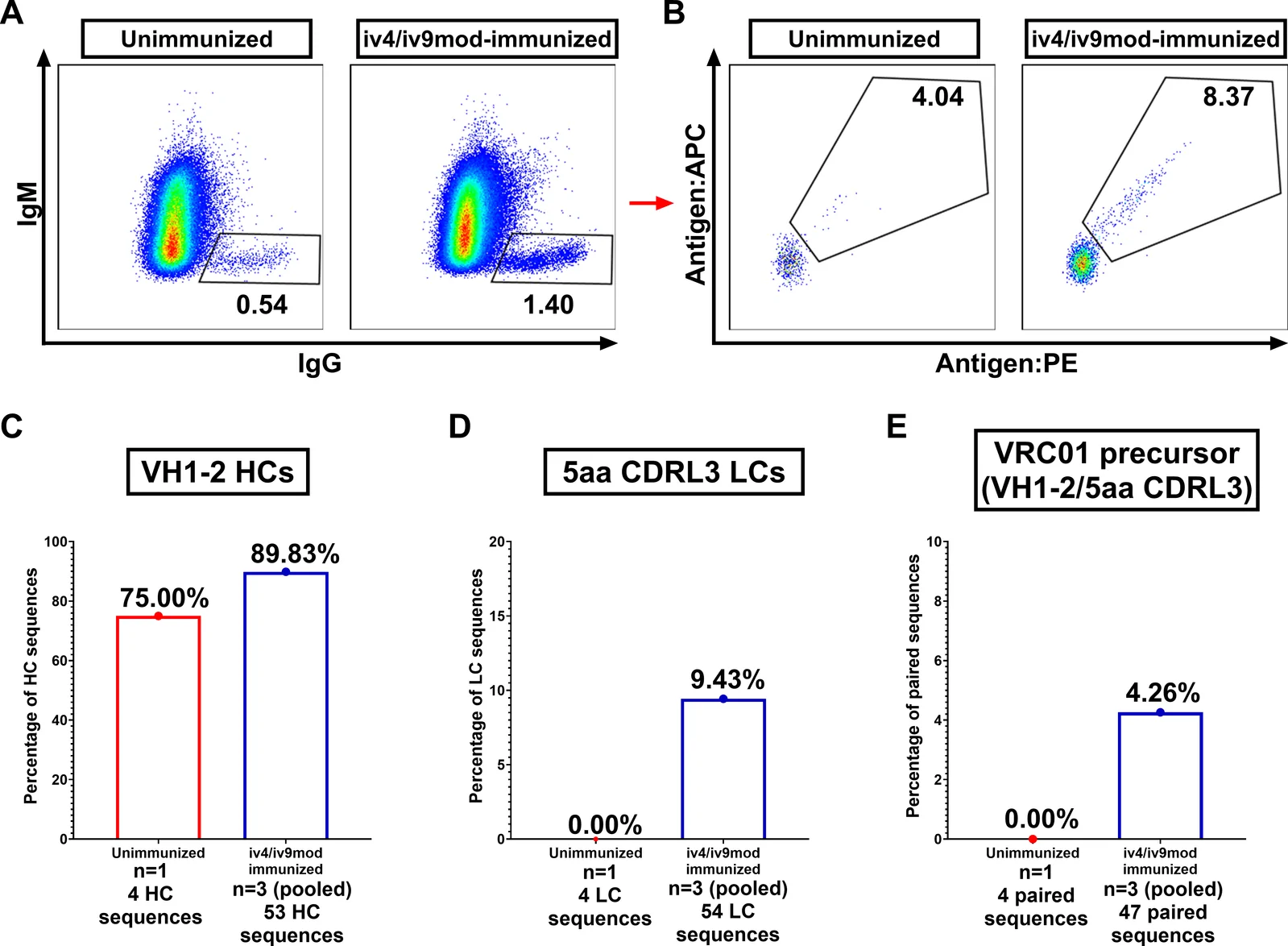
Single-cell sorting and sequencing of antigen-specific B cells from ATX-GK mice immunized with lower-affinity ai-mAb. (**A**) Frequency of IgG^+^ B cells from lymph nodes of iv4/iv9mod-immunized or unimmunized ATX-GK mice as indicated. (**B**) Frequency of antigen-specific B cells among lymph node IgG^+^ B cells in iv4/iv9mod-immunized or unimmunized ATX-GK mice as indicated. (**C–E**) Single, antigen-specific IgG^+^ lymph node B cells were sorted from unimmunized (red bars) or iv4/iv9mod-immunized ATX-GK mice (blue bars). Sanger sequencing was used to determine frequencies of VH1-2 heavy chains (**C**), 5aa CDRL3 LCs (**D**), and VRC01 precursors (**E**).

To identify antigen-specific B cells, we used iv4/iv9mod as bait to single-cell sort and sequence 89 IgG^+^ splenic B cells from mice #9 and #10 (Fig. S2, Tables S3 and S6). We identified a high frequency of VH1-2 HCs (≥95%) from antigen-specific splenic B cells in both mice but were unable to identify any 5aa CDRL3 LCs (Tables S5 and S6). Next, we single-cell sorted and sequenced antigen-specific IgG^+^ B cells pooled from lymph nodes collected from the three iv4/iv9mod-immunized mice (mice #9–#11, Tables S3, S5 and S6) and an unimmunized mouse (mouse #4, Tables S3, S5 and S6). In this compartment, we observed a higher frequency of IgG^+^ B cells in immunized mice compared to the unimmunized mouse (Fig. 4A). Among these, the frequency of antigen-specific IgG^+^ B cells was twofold higher in the immunized sample compared to the unimmunized sample (Fig. 4B). The frequency of B cells expressing VH1-2 HCs among antigen-specific cells was slightly higher in the immunized sample than the unimmunized sample (89.83% vs 75.00%- Fig. 4C). No antigen-specific B cells sorted from unimmunized mice expressed 5aa CDRL3 LCs, while 9.43% did in the immunized sample (Fig. 4D). Compared to unbiased sequencing of naïve mice, this frequency of 5aa CDRL3 LCs is 176-fold higher (Fig. 1B). We identified two putative VRC01 precursors (PATXP4 and PATXP5) among sorted lymph node B cells from the iv4/iv9mod-immunized group and none from the unimmunized mouse (Fig. 4E; Tables S4 to S6). Based on CDRH3 sequences, CDRL3 sequences, and light-chain gene usage, PATXP4 and PATXP5 were clonal variants (Table S4). PATXP4 and PATXP5 also appear to be clonal relatives of one of the VRC01 precursors, PATXP3, identified among sorted IgG^+^ splenic B cells, which presumably arose from either mouse #9 or mouse #10 that were common to both analyses (Fig. 3D and 4E; Tables S4 to Table S6).

### Immunization with an anti-idiotypic immunogen in three additional ATX-GK mice did not lead to identification of VRC01 precursors

**Fig 5:**
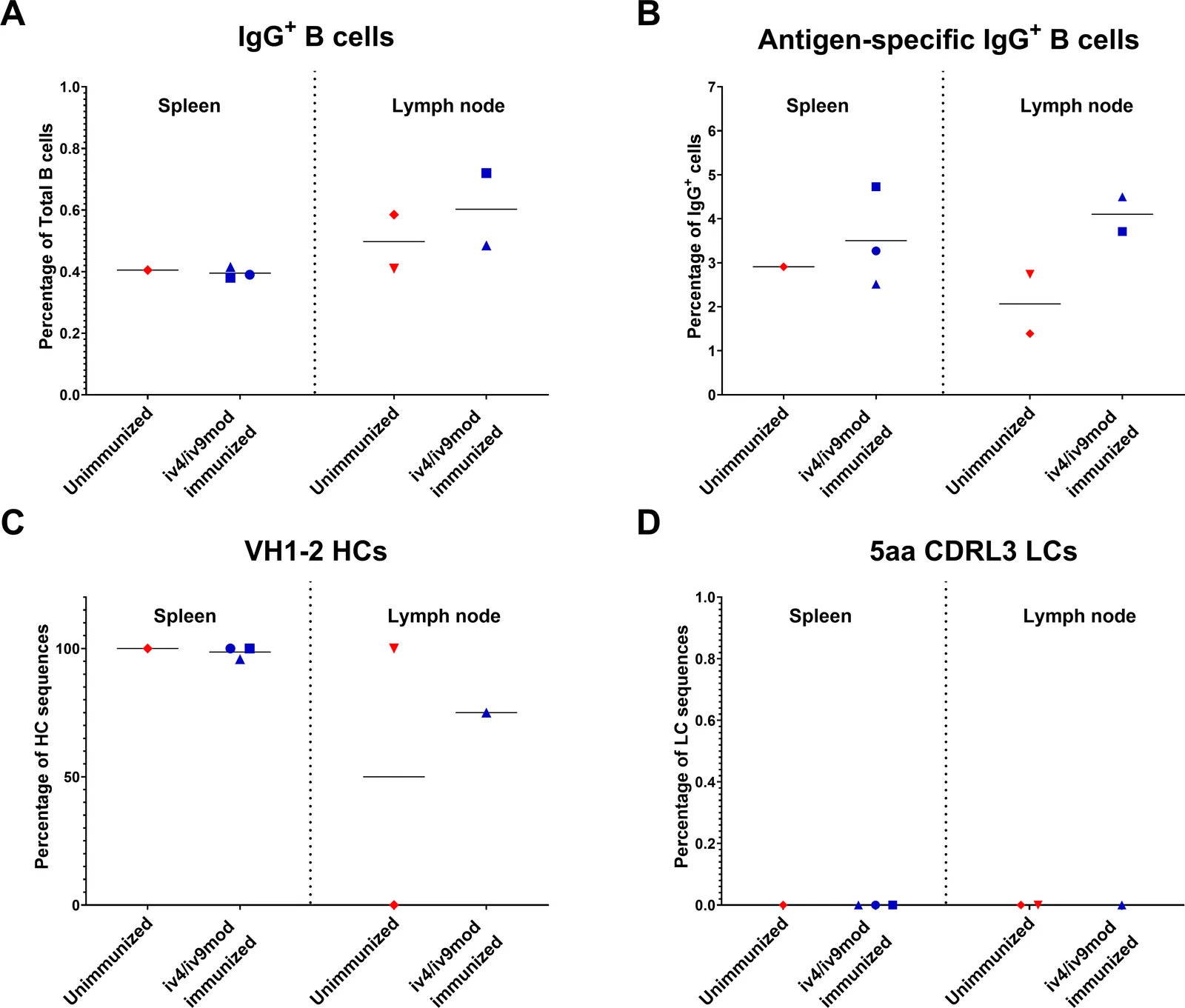
Single-cell sorting and sequencing of B cells from the spleens and lymph nodes of ATX-GK mice immunized with lower-affinity ai-mAb. (**A–D**) Cells from either spleens or lymph nodes of unimmunized and iv4/iv9mod-immunized mice were sorted using iv4/iv9mod antigen and sequenced. Each symbol represents an individual mouse across all graphs. (**A**) Percentage of IgG^+^ B cells among total B cells as indicated. (**B**) Percentage of iv4/iv9mod-specific cells among IgG^+^ B cells from panel **A** as indicated. (**C**) Percentage of VH1-2 heavy-chain sequences derived from iv4/iv9mod-specific cells from panel **B** as indicated. (**D**) Percentage of light chains with 5aa CDRL3s among total light-chain sequences derived from iv4/iv9mod-specific cells from panel **B** as indicated.

To determine if VRC01 precursors are reproducibly detectable in additional mice, we repeated the iv4/iv9mod immunizations in three additional ATX-GK mice (Table S3). Here, we sorted splenocytes and lymph nodes from individual mice separately. We performed antigen-specific sorting followed by RT-PCR and Sanger sequencing of VH and VL transcripts on B cells from lymph nodes and spleens of both unimmunized and immunized mice. Overall, we sorted splenic B cells from unimmunized mouse #6 and three iv4/iv9mod-immunized mice (mice #12–#14, Tables S5 and S6). Additionally, we sorted B cells from lymph nodes of two unimmunized (mice #5 and #6) and two iv4/iv9mod-immunized mice (mice #13 and #14, Tables S5 and S6). We were unable to recover any amplicons from antigen-specific B cells sorted from the lymph nodes of mouse #13 of the iv4/iv9mod-immunized group, which was excluded from sequencing analysis (Fig. 5, blue squares and Tables S5 and S6).

There was no difference in the frequency of IgG^+^ splenic B cells between unimmunized vs iv4/iv9mod-immunized mice (Fig. 5A). However, there was a slightly higher frequency of IgG^+^ B cells in the lymph nodes of immunized mice (Fig. 5A). There were higher frequencies of antigen-specific IgG^+^ B cells in both the spleen and lymph nodes of immunized mice compared to unimmunized mice (Fig. 5B). The frequency of VH1-2 HCs was enriched among antigen-specific IgG^+^ B cells from both spleen and lymph nodes from either group compared to baseline frequencies (Fig. 1A and 5C). However, we were unable to identify any light-chain sequences with 5aa CDRL3s in either group (Fig. 5D). To confirm that mice responded to the immunization, we performed ELISAs to assess the antigen-specific binding of sera pre- and post-immunization. Binding titers to iv4/iv9mod were higher 14 days post-immunization compared to baseline in all mice (Fig. S3). In sum, we were unable to identify any VRC01 precursors from 115 cells sorted from 4 million B cells from both lymph nodes and spleens of the second cohort of three iv4/iv9mod-immunized mice (Tables S5 and S6). Although it should be noted that the heavy- and light-chain transcript recovery was not 100% efficient (Tables S5 and S6). Overall, we detected VRC01 precursor B cells in the initial cohort of three mice, but none were detected in the second cohort of three individually analyzed iv4/iv9mod-immunized mice.

### Putative VRC01 precursors isolated from ATX-GK mice do not bind to germline-targeting, Env-derived proteins

**Fig 6:**
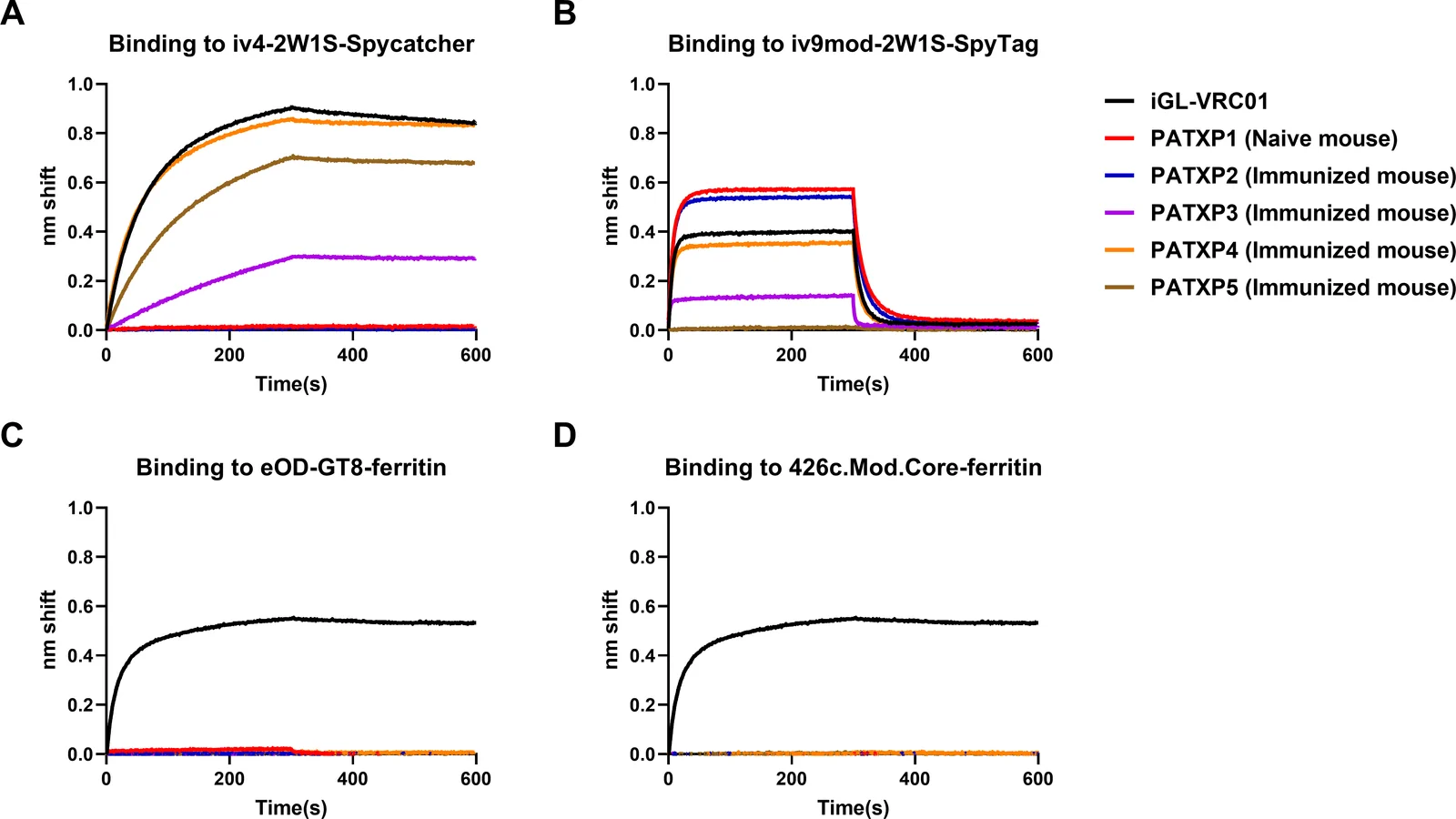
Identified putative VRC01 precursors do not bind to Env-derived immunogens. The indicated antibodies isolated from ATX-GK mice (denoted by the PATXP prefix) were immobilized on biosensors and then immersed in a solution with iv4-2W1S-Spycatcher at 250 nM (**A**), iv9mod-2W1S-Spytag at 250 nM (**B**), eOD-GT8-ferritin at 1 µM (**C**), and 426c.Mod.Core-ferritin at 1 µM (**D**).

To assess if the VRC01-class precursors identified from ATX-GK mice could bind a more native-like Env-derived immunogen, we produced the VRC01-class precursor isolated from a naïve ATX-GK mouse (PATXP1) as well as the four putative VRC01 precursors (PATXP2–PATXP5) isolated from iv4/iv9mod-immunized mice as recombinant mAbs (47). We tested binding to monomeric iv4 Fab, monomeric iv9mod Fab, and two multimeric, germline-targeting Env-derived proteins: eOD-GT8-ferritin (34) and 426c.Mod.Core-ferritin (33). PATXP3 (purple curve) and PATXP4 (orange curve) from the immunized mice bound to both components of the bispecific ai-mAb (Fig. 6A and B). PATXP5 (brown curve) from the immunized mice only bound to iv4. PATXP1 (red curve) from the naïve mouse and PATXP2 (blue curve) from the immunized mice only bound to iv9mod. However, no mAbs bound to either germline-targeting Env-derivatives (Fig. 6C and D).

## DISCUSSION

Here, we assessed whether ATX-GK mice from Alloy Therapeutics could model the human antibody repertoire and serve as a surrogate for evaluating the ability of germline-targeting immunogens derived from ai-mAbs to prime VRC01 class precursor B cells. From our deep-sequencing analysis of splenic B cells, we found that ATX-GK mice express the VH1-2 heavy chain at near human physiological frequency (~3%). However, these mice express a 5aa CDRL3 light chain at ~8-fold lower frequency than found in humans, resulting in a lower expected frequency of VRC01-class B cells in ATX GK mice as compared to humans. Murine CDRL3s are less diverse than those found in humans and are typically 9aa long. Terminal deoxynucleotidyl transferase (TdT) increases VDJ junction diversity through non-templated nucleotide additions to the 3′ end of DNA, as well as suppresses recurrent CDR3s resulting from microhomology-mediated VDJ joining (48–50). TdT is expressed during light-chain VDJ recombination in postnatal human pre-B cells, but not in postnatal murine pre-B cells (50–52). The limited diversity in CDRL3 length in ATX-GK mice is consistent with a lack of TdT activity during light-chain recombination (39). A similar low frequency of 5aa CDRL3s was observed in an alternative murine model with a transgenic, genetically human, rearranging antibody repertoire, which also harbored frequencies of VRC01-class B cells that were lower than those in humans (53). Alternative transgenic mouse strains that co-express rearranging VH1-2 HCs and human LCs, along with the human TdT enzyme, may harbor more relevant frequencies of VRC01 precursors than ATX-GK mice for evaluating germline-targeting immunogens (50, 54). Further engineering of ATX-GK mice to express TdT during light-chain recombination may be a strategy to increase CDRL3 diversity, yield antibody light-chain sequences more similar to those found in humans, and increase the VRC01-class precursor frequency.

Despite the paucity of VRC01-class precursor B cells, we identified precursor VRC01-class splenic B cells in naïve ATX-GK mice using iv4/iv9-baited B-cell sorting. However, we also isolated an excess of non-VRC01-class B cells with VH1-2 heavy chains, which could potentially out-compete VRC01-class precursors in the context of immunization (38, 53, 55, 56). In an attempt to limit this, we leveraged structure-guided design to lower the affinity of the iv9 arm for VH1-2-derived heavy chains. This strategy appeared to increase the specificity for VRC01 precursors, as evidenced by detection of class-switched VRC01-class precursors following immunization with iv4/iv9mod but not with iv4/iv9; however, this may be a spurious finding, as VRC01-class precursors were only observed in a minority of mice, and the overall animal numbers were small in this study. A large proportion of off-target cells with VH1-2 heavy chains but irrelevant light chains were still activated by iv4/iv9mod, suggesting further optimization could be possible. For example, we could try improving the affinity of iv4 for binding to 5aa CDRL3 light chains through high-throughput yeast-display mutagenesis (57), deep mutational scanning (58, 59), or other machine learning techniques (60, 61). These techniques could be an effective means to improve the specificity of the bispecific molecule.

It has been proposed that a multi-stage vaccination scheme with an immunogen that can target VRC01-class precursors (germline-targeting), followed by boosting with more native-like Env derivatives, will be necessary to drive somatic mutation and elicit potently neutralizing VRC01-class bNAbs (27–33). Of the three VRC01 precursors identified from ai-mAb-immunized ATX-GK mice, none bound to eOD-GT8-ferritin nor to 426c.Mod.Core-ferritin, suggesting that the putative VRC01-class precursors elicited by the ai-mAb would not be boosted by these immunogens. The absence of binding of the mAbs to these two germline-targeting immunogens is not entirely unexpected, since germline-targeting Env immunogens do not bind all BCRs that meet the minimum definition of a VRC01-class precursor, that is, VH1-2 HC/5aa CDRL3 LC paired BCRs (38, 62, 63). Indeed, among three putative VRC01 precursors isolated by iv4/iv9 sorting of naïve human B cells, one bound to eOD-GT8, and none bound to 426c.Mod.Core (34). Furthermore, we previously showed that although immunization with iv4/iv9 could activate iGL-VRC01-class B cells in an adoptive transfer model, the immunogen drove somatic mutation away from Env recognition (36). A similar phenomenon may have selected for similar off-track mutations in VRC01-class BCRs in the ATX-GK mice studied here.

ATX-GK mice harbor rare VRC01-class precursor-like B cells, but this model presents substantial challenges for reliable targeting by the anti-idiotype-derived immunogen described herein. We speculate that the ai-mAb could be more successful in humans, where the frequencies of VRC01-class precursors are higher. Future studies that compare the B-cell responses elicited by the ai-mAb and Env-derived germline-targeting immunogens in ATX-GK mice could inform whether this mouse model is a useful surrogate for human immunization, which would enable a means of evaluating prime-boost regimens in a small animal model. Limitations of our study include the use of a limited number of mice and potential under-sampling of the B cell compartment, which may have led to an inability to detect VRC01 precursor B cells.

## MATERIALS AND METHODS

### Mice

Mice were purchased from Alloy Therapeutics and shipped from Charles River Laboratories (Wilmington, MA, USA) (64). All mice used in our studies were housed with free access to food and water, with a 12:12 light:dark cycle. Immunizations and retro-orbital bleeds were carried out under anesthesia, which was induced by administering isoflurane, set at 1–5% for 1–2 min in an induction chamber with the flow rate of O_2_ set at 1.0 L/min. Animals under anesthesia were then transferred to a nose cone and continued to receive 1–5% isoflurane at an O_2_ set to 1.0 L/min during injections and retro-orbital bleeds. Mice were euthanized by administering 100% CO_2_ in an induction chamber with a flow rate of 3.0 L/min to allow for 50% of the air in the chamber to be replaced per minute for at least 5 min, followed by cervical dislocation.

### Germline reversion of iv9

The iv9 HC with the V region reverted to the murine IGH1-59*01 V gene sequence, and the iv9 LC with the V region reverted to the murine IGKV8-28*01 V gene sequence were ordered as human codon-optimized gBlocks synthetic gene fragments (Integrated DNA Technologies) with adapters for cloning (65). The VH/VL sequences were cloned into full-length pTT3-derived IgG1 and IgK expression vectors containing human constant regions using Gibson assembly (47). Vectors were transformed into DH5-alpha cells (NEB) and plasmid sequencing was performed (Azenta Life Sciences).

#### Site-directed mutagenesis

Site-directed mutagenesis to add back D52W_HC_ and H92E_LC_ to germline iv9 was performed using the Agilent QuikChange Primer Design online tool and Agilent QuikChange II Site-Directed Mutagenesis Kit (Agilent, Cat. #200523) according to the manufacturer’s instructions. Vectors were transformed into DH5-alpha cells (NEB) and plasmid sequencing was performed (Azenta Life Sciences).

Primer sequences were as follows: D52W_HC_ Forward primer, 5′-aagagtcggagggccatattacaccgatccattccagcccc-3′; D52W_HC_ Reverse primer, 5′-ggggctggaatggatcggtgtaatatggccctccgactctt-3′; H92E_LC_ Forward primer, 5′-caaaggtgtatggataggactcatcattctggcaataataaactgcc-3′; H92E_LC_ Reverse primer, 5′-ggcagtttattattgccagaatgatgagtcctatccatacacctttg-3′.

### Plasmids

pTT3-iv4-Fab-HC-SC, pTT3-iv4-LC-2W1S, pTT3-iv9-Fab-HC-ST, and pTT3-iv9-LC-2W1S were previously described (34, 36). The mAb leader sequence and VH regions of iGL-iv9 D52W were also cloned into the Fab-Spytag construct (pTT3-iGL-iv9 D52W-Fab-HC-ST). The mAb leader sequence and VL regions of iGL-iv9 H92E were also cloned into the Fab-2W1S LC construct (pTT3-iGL-iv9 H92E-2W1S-LC).

iGL-VRC01 (VK3-11-derived kappa chain with C98S mutation in the heavy chain) was prepared as previously described (8, 26, 28, 66).

pTT3-426c TM4Δ1-3-ferritin (426.Mod.Core-ferritin) (33) and pTT3-eOD-GT8-ferritin (34) were previously described.

### IgG expression and purification

All recombinant IgGs were produced in 293E cells in Freestyle 293 media using the 293Free transfection reagent according to the manufacturer’s instructions. The cells were transfected at a density of 10^6^ cells/mL. Heavy-chain-encoding (250 µg) and light-chain-encoding (250 µg) encoding plasmids were co-transfected per liter of suspended culture. Expression was carried out for 6 days, after which cells and cellular debris were removed by centrifugation at 4,000 × *g*, followed by filtration through a 0.22 µm filter. Clarified cell supernatant containing recombinant antibodies was passed over protein A agarose resin. Protein A resin was extensively washed with PBS and eluted with IgG elution buffer. Fractions containing purified IgG were pooled, buffer exchanged into PBS, aliquoted, frozen in liquid nitrogen, and stored at −20°C.

### SpyCatcher/SpyTag Fab purification

pTT3-iv4-Fab-HC-SC plus pTT3-iv4-2W1S LC and pTT3-iv9-Fab-HC-ST (or pTT3-iGL-iv9 D52W-Fab-HC-ST) plus pTT3-iv9-2W1S-LC (or pTT3-iv9 H92E-2W1S-LC) were transfected into 293E cells at a density of 10^6^ cells/mL in Freestyle 293 media using the 293Free transfection reagent according to the manufacturer’s instructions. Expression was carried out for 6 days, after which cells and cellular debris were removed by centrifugation at 4,000 × *g*, followed by filtration through a 0.22 µm filter. Clarified cell supernatant containing iv4-2W1S-SC, iv9-2W1S-ST, or iGL-iv9 D52W H92E-ST was passed over Ni-NTA resin, pre-equilibrated with Ni-NTA binding buffer (0.3 M NaCl, 20 mM Tris, and 10 mM imidazole, pH 8.0), followed by extensive washing with Ni-NTA binding buffer, and then eluted with 250 mM imidazole, 0.3 M NaCl, 20 mM Tris, pH 8.0 (Ni-NTA elution buffer). Proteins were further purified by SEC using an Enrich SEC 650 10 × 300 column equilibrated in PBS. To generate bispecific iv4/iv9 or iv4/iGL-iv9 D52W H92E, iv4-Fab-SC was incubated with a 1.5-fold excess of iv9-Fab-ST or iGL-iv9 D52W H92E-ST for 1 h at room temperature (RT). The complex was then separated from excess iv9-Fab-ST or iGL-iv9 D52W H92E-Fab-ST using an Enrich SEC 650 10 × 300 column equilibrated in PBS. iv4/iv9 and iv4/iGL-iv9 D52W H92E were either conjugated to DyLight 550-NHS ester or DyLight-650-NHS ester (Thermofisher) and then flash-frozen or were flash-frozen without a fluorescent label and stored at −20°C.

### Calcium flux assays

Calcium mobilization in B cells stably expressing iGL-VRC01 BCRs upon antigenic stimulation was monitored as previously described (26, 28, 34). Briefly, prior to antigenic challenge, the cells were loaded with Fluo-4 Direct calcium indicator, mixed 1:1 with complete RPMI at 37°C for 1 h. The cells were pelleted and stained with PE-conjugated anti-human Fcγ Fab (Jackson ImmunoResearch) (1:100 dilution in 100 μL complete RPMI plus Fluo-4 Direct) for 30 min. The cells were washed with 5 mL of complete RPMI and resuspended at ~2 × 10^6^ cells/mL in complete RPMI and subjected to Ca^2+^ flux analysis at a medium flow rate on a FACSymphony A5 (BD Biosciences) flow cytometer. In all cases, BCR transduced cells were mixed with untransduced cells as an internal control at a 5:2 ratio. Levels of background fluorescence (Min_FL_) were determined by averaging the background Fluo-4 absorbance in cells for 30 s. After that, activation of B cells expressing exogenous BCRs by the various immunogens was determined by monitoring changes in Fluo-4 fluorescence associated with cells expressing the exogenous BCRs (PE^+^ cells) for 210 s. The bispecific iv4/iGL-iv9 D52W H92E anti-idiotype, iGL-iv9 D52W H92E-ST Fab, iv4-SC Fab, a control mAb (anti-EBV AMMO1 [42]) or 426c.Mod.Core-ferritin were added at a final concentration of 100 nM. Ionomycin was added to a final concentration of 150 nM for 60 s following the immunogen addition, and maximum Fluo-4 fluorescence (Max_FL_) was established by averaging the Fluo-4 fluorescence signal recorded during the last 10 s. The percent of maximum Fluo-4 fluorescence at each time point *t* was determined using the following formula: (fluorescence at *t* − Min_FL_)/(Max_FL_ − Min_FL_) × 100. This analysis was performed on both the BCR-positive (anti-human Fcγ-PE^+^) and BCR-negative cells (anti-human Fcγ-PE^-^) simultaneously. The background Fluo-4 fluorescence signal from the BCR-negative cells was subtracted from that of the BCR-positive population at each time point. All flow analysis was done with FlowJo 10.8.1 software (BD).

### Biolayer interferometry (BLI)

BLI assays were performed on the Octet Red 96E Instrument (Sartorius) at 30°C with shaking at 1,000 RPM.

#### iv9 mutants binding screen

iv9 IgG and iGL-iv9 IgG mutated derivatives were immobilized on AHC biosensors for 150 s. After loading, the baseline signal was then recorded for 60 s in KB. The sensors were then immersed in wells containing iGL-VRC01 Fab at 250 nM for 300 s (association phase), followed by immersion in KB for an additional 300 s (dissociation phase).

#### Kinetics analysis

iGL-iv9 D52W H92E IgG was immobilized on anti-human IgG Fc capture (AHC) biosensors (Sartorius) for 150 s. Sensors were immersed in KB for 30 s to collect a baseline measurement. Sensors were then immersed in wells containing twofold serial dilutions of iGL-VRC01 Fabs in 1× KB buffer, and the association was measured for 300 s, followed by immersion in 1× KB for 600 s to measure dissociation. Empty reference sensors were used to measure the background signal from each analyte-containing well. Additionally, each ligand-coupled sensor was associated in wells containing only buffer as a reference. Double reference subtraction was performed for each corresponding ligand-coupled sensor at each time point. Curve fitting was performed using a 1:1 binding model in Octet BLI Analysis 12.2 software. Mean *k*_dis_ (1/s) and *k*_on_ (1/Ms) values were determined by averaging all binding curves that matched the theoretical fit with an *R*^2^ value of ≥0.99. The *K*_*D*_ value was calculated by taking the mean *k*_dis_ value divided by the mean *k*_on_ value.

#### Bispecific anti-idiotype binding screen

iGL-VRC01 IgG and chimeric IgGs, comprised of either a VRC01-class IgH paired with a non-VRC01-class IgL or a non-VRC01-class IgH paired with a VRC01-class IgL (28), were immobilized on AHC biosensors for 150 s. After loading, the baseline signal was recorded for 60 s in KB. The sensors were then immersed into wells containing iv4/iGL-iv9 D52W H92E at 250 nM for 300 s (association phase), followed by immersion in KB for an additional 300 s (dissociation phase).

#### Binding screens with putative VRC01 precursors from naive human B cells

mAbs (34, 40) were immobilized on AHC biosensors for 150 s. After loading, the baseline signal was then recorded for 60 s in KB. The sensors were then immersed in wells containing 250 nM iv4/iv9 or 250 nM iv4/iv9mod diluted in KB for 300 s (association phase), followed by immersion in KB for an additional 300 s (dissociation phase).

#### Binding screens with putative VRC01 precursors from ATX-GK mice

mAbs were immobilized on AHC biosensors for 150 s. After loading, the baseline signal was then recorded for 60 s in KB. The sensors were then immersed in wells containing various proteins (iv4-2W1S-SC and iGL-iv9 D52W H92E-2W1S-ST at 250 nM; eOD-GT8-ferritin and 426c.Mod.Core-ferritin at 1 μM) diluted in KB for 300 s (association phase), followed by immersion in KB for an additional 300 s (dissociation phase).

### Immunizations

Mice were pre-primed with 40 micrograms of 2W1s peptide mixed with 5 micrograms of lipopolysaccharide via retro-orbital injection behind the right eye. Fourteen days later, ten micrograms of iv4/iv9-2W1s or iv4/iGL-iv9 D52W H92E-2W1S was resuspended in 50 μL PBS mixed with 50 μL of SMNP (45) adjuvant and injected intramuscularly split between each hind leg. This route was selected to be consistent with previous murine studies evaluating these molecules (34, 36). Mice were euthanized 14 days after immunization, where spleens and lymph nodes (inguinal, axillary, and lumbar) were harvested and whole blood was collected by terminal cardiac bleed. Single-cell suspensions were prepared by mechanical disruption of the spleens or lymph nodes through a 70 μm filter and incubating with 2 mL of RBC lysing buffer. The cells were quenched with FACS buffer then resuspended in 10% DMSO/90% FBS at 5M or 20M/mL tubes. The cells were frozen in quick-freeze cell containers (Thermo Scientific) overnight at −80°C before transfer to liquid nitrogen storage. Serum was obtained by subsequent centrifugation at 9,600 × *g* for 5 min, heat-inactivated at 56°C for 10 min, then stored at 4°C.

### Antigen-specific serum IgG ELISA

Microplages (384 wells) were coated with 60 ng/well iv4/iGL-iv9 D52W H92E in 0.1 M NaHCO_3_ pH 9.4–9.6 (ELISA coating buffer) at RT overnight at 30 μL/well. The next day, plates were washed four times with 1× PBS and 0.02% Tween 20 (ELISA wash buffer) prior to blocking for 1 h with 100 μL/well of PBS containing 10% non-fat milk and 0.02% Tween 20 (ELISA blocking buffer) at 37°C. After blocking, serial dilutions of serum in ELISA blocking buffer were added and incubated for 1 h at 37°C. 32 control wells were included that contained only blocking buffer without sera or mAb standard. Plates were incubated for 1 h at 37°C. After washing, a 1:10,000 dilution of Peroxidase AffiniPure Goat Anti-Mouse IgG, Fcγ fragment specific (Jackson ImmunoResearch, Cat. #115-035-071) in ELISA blocking buffer was added to each well and incubated 1 h at 37°C. After four washes, 30 μL/well of SureBlue Reserve TMB Microwell peroxidase substrate (SeraCare, Cat. #5120-0081) was added. After 5 min at room temperature, 30 μL/well of 1 N sulfuric acid was added and the *A*_450_ of each well was read on a Molecular Devices SpectraMax M2 plate reader. The average *A*_450_ values of buffer-only control wells were subtracted from each serum-containing well and plotted in GraphPad Prism 10. *A*_450_ values were plotted as a function of the log_10_ of the serum dilution. A binding curve was fit using the sigmoidal, 4PL, *X* is log(concentration) least squares fit function.

### B-cell sorting

B cells from ATX-GK mice were enriched from either cryopreserved single-cell suspensions from spleens or lymph nodes using the EasySep Mouse B cell isolation kit (StemCell, Cat. #19854). CD16/CD32 Fc block (BioLegend, Cat. #101302) was added at 1:200 dilution for 15 min at RT. The cells were stained using a BV510 viability dye (eBioscience, Cat. #65-0866-14) at 1:200 dilution, and the following antibodies at a 1:100 dilution unless otherwise noted: CD3 BV711 (BioLegend, Cat. #100349), Gr-1 BV711 (BioLegend, Cat. #127643), F4/80 BV711 (BioLegend, Cat. #123147), CD19 BUV395 (BD, Cat. #563557; 1:200 dilution), B220 BV786 (BioLegend, Cat. #103246; 1:250 dilution), IgM Superbright 600 (eBioscience, Cat. #63-5790-82; 1:200 dilution), and IgG FITC (Jackson ImmunoResearch, Cat. #115-095-164; 1:200 dilution). Dylight-labeled antigens were added at 0.5 μg per sample of cells. The cells were stained for 30 min on ice, washed twice in FACS buffer, and resuspended in 200 μL sorting buffer (PBS + 2% BSA) for sorting and analysis on a BDFACS Symphony S6. The cells were single-cell sorted into 96-well plates using the following gating strategy: lymphocytes/singlets/live, CD3^−^, Gr-1^−^, F4/80^−^/CD19^+^B220^+^ (B cells)/ IgM^−^, and IgG^+^/antigen^++^.

### Single-cell BCR sequencing

#### 10× sequencing of naive ATX-GK mice

B cells were enriched from splenocytes of two naive ATX-GK mice independently using the EasySep Mouse B cell kit (StemCell, Cat. #19854) according to the manufacturer’s instructions. Paired BCR sequencing of B cells was performed using the Chromium Next GEM Single Cell 5′ Kit v2 (10× Genomics, Cat #. PN-1000265). 16,500 B cells were input to generate gel beads-in-emulsions (GEMs) for each mouse. Custom primers from Alloy Therapeutics for IgM and IgK chains were utilized to amplify VH and VL sequences (39). Library sequencing was performed on a NextSeq 2000 (Illumina) by the Fred Hutchinson Cancer Center Genomics and Bioinformatics Core Facility (RRID:SCR_022606). The BCR libraries were then mapped to a human VDJ reference using Cell Ranger (10× Genomics). Each paired BCR’s V, D, and J genes for both heavy chains and light chains were determined utilizing Enclone (10× Genomics).

#### 10× sequencing of IgG^+^ B cells from ATX-GK mice

IgG^+^ B cells were sorted from ATX-GK mice (see B-cell sorting methods above). Paired BCR sequencing of B cells was performed using the Chromium Next GEM Single Cell 5× Kit v2 (10× Genomics, Cat. #PN-1000265). Sorted B cells were inputted to generate gel beads in emulsions (GEMs) for each mouse. Custom primers from Alloy Therapeutics for IgG and IgK chains were utilized to amplify VH and VL sequences. Library sequencing was performed on a NextSeq 2000 (Illumina) by the Fred Hutch Cancer Center Genomics and Bioinformatics Core Facility (RRID:SCR_022606). The BCR libraries were then mapped to a human VDJ reference using Cell Ranger (10× Genomics). The V, D, and J genes for each paired BCR were determined utilizing Enclone (10× Genomics).

#### Sanger sequencing of antigen-specific IgG^+^ B cells from ATX-GK mice

Antigen-specific IgG^+^ B cells were single-cell sorted into 96-well plates. cDNA was generated using SuperScript IV First-Strand Synthesis System (Invitrogen, Cat. #18091050). The VH and VL sequences were recovered using gene-specific human forward primers, custom ATX-GK reverse primers, and cycling conditions previously described (47). VH or VL amplicons were purified using the Monarch PCR & DNA Cleanup Kit (NEB, Cat. #T1030L) and Sanger sequenced (Azenta Life Sciences). The antibody gene usage was assigned using IMGT VQUEST (65).

#### Generation of putative VRC01 precursor mAbs

Putative precursor VRC01 VDJ sequences identified from sorting and sequencing were ordered as human codon-optimized gBlocks synthetic gene fragments (IDT) with adapters for cloning into pTT3 plasmids. The VH/VL sequences were cloned into full-length pTT3-derived IgG1 and IgK expression vectors containing human constant regions using Gibson assembly (47). Vectors were transformed into DH5alpha cells (NEB), and plasmid sequencing was performed (Azenta Life Sciences). mAbs were produced from transfections of 293E cells as described above.

## Data Availability

Sequences of the PATXP1–PAXTP5 mAbs have been deposited in NCBI GenBank under accession numbers PZ620292–PZ620301. BCR sequences from iv4/iv9-sorted naïve ATX-GK mice and from IgG^+^ sorted B cells from iv4/iv9-immunized and iv4/iv9mod-immunized mice obtained by microfluidics emulsion-based single-cell RNA sequencing data have been deposited under BioProject PRJNA1484700. BCR heavy- and light-chain sequences of iv4/iv9mod-specific single-cell sorted IgG^+^ B cells from unimmunized and iv4/iv9mod-immunized ATX-GK mice are provided in Table S6.

## SUPPLEMENTAL MATERIAL

Supplemental Material msphere.00317-26-s0001.pdf Fig. S1 to S3; Tables S1 to S5.

Table S6 msphere.00317-26-s0002.xlsx Sequences of iv4/iv9mod-sorted B cells from unimmunized and iv4/iv9mod-immunized ATX-GK mice.

ASM does not own the copyrights to Supplemental Material that may be linked to, or accessed through, an article. The authors have granted ASM a non-exclusive, world-wide license to publish the Supplemental Material files. Please contact the corresponding author directly for reuse.
