# Supplementary material for: Evaluating an anti-idiotype derived germline-targeting immunogen designed to elicit VRC01-class precursors in human antibody transgenic mice
Source: mSphere. 2026 Sep 1;11(9):e00317-26. doi: 10.1128/msphere.00317-26 (PMC13621863; doi:10.1128/msphere.00317-26)
Supplement: Supplementary file 1 [file msphere.00317-26-s0001.pdf]

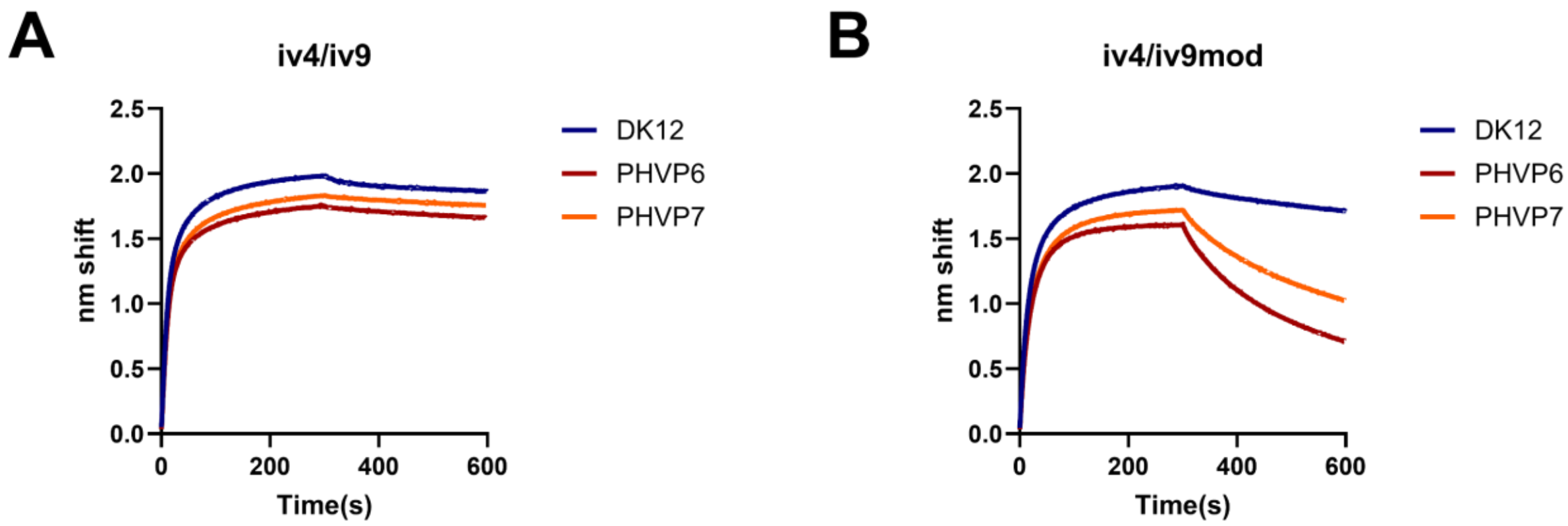

Supplementary Figure 1. iv4/iv9mod binds to naturally paired putative VRC01-class antibodies isolated from naïve human B cells

Binding of iv4/iv9 (**A**) and iv4/iv9mod (**B**) to previously isolated naturally paired putative VRC01-class antibodies (defined as VH1-2 HC paired with 5aa CDRL3 LC) isolated from naïve human B cells was measured by BLI. PHVP6 and PHVP7 were isolated from naïve human PBMCs sorted using iv4/iv9[34]. DK12 was identified by unbiased high-throughput sequencing[40].

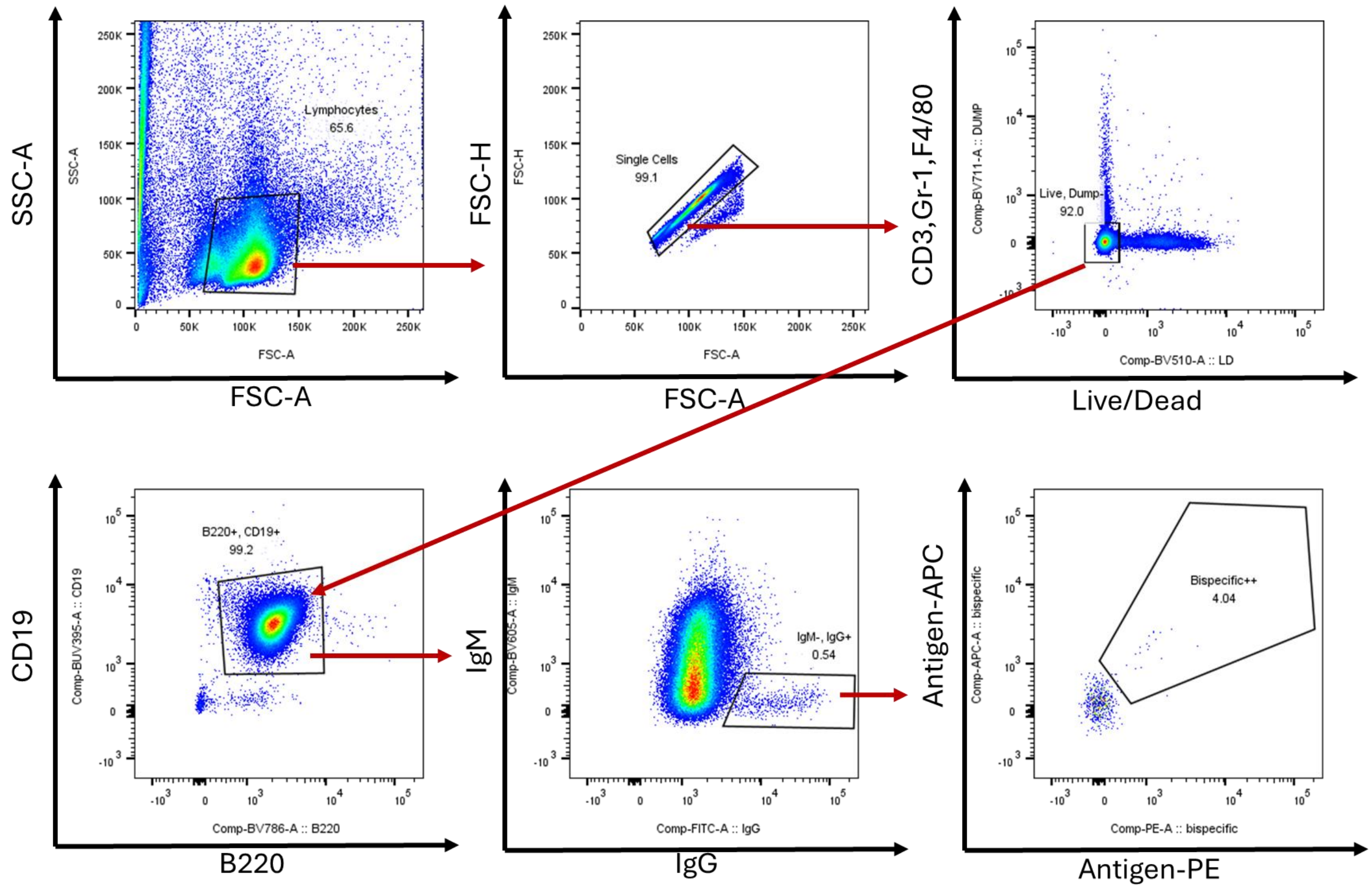

Supplementary Figure 2. Representative gating strategy for IgG+ antigen-specific B cell sorting

B cells from spleens or lymph nodes were enriched using a murine B cell isolation kit then sorted based on the following gate strategy: lymphocytes > Single cells > Live cells > $CD19^{+}B220^{+}$ > $IgM^{-}IgG^{+}$. For antigen-specific sorts, IgG^{+} cells were further gated on $antigen^{++}$ cells.

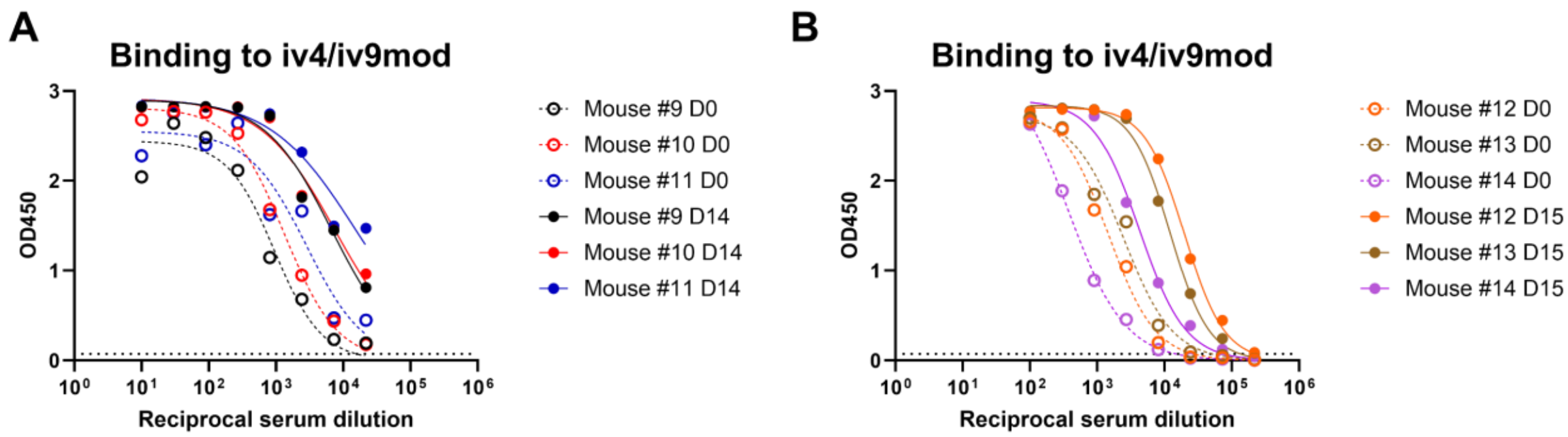

Supplementary Figure 3. **Pre- and post-immunization sera binding to iv4/iv9mod anti-idiotypic immunogen.** Antigen-specific ELISA was performed using sera from the first iv4/iv9mod immunization study (**A**) and repeat study (**B**).

Table S1: Expected number of VRC01 precursors in an individual naïve ATX-GK mice. Related to Figure 1.

Mouse ID	VH1-2 HC frequency (%)	5aa CDRL3 LC frequency (%)	Expected frequency of VRC01 precursors (%)	Expected number of VRC01 precursors*
Naïve Mouse #1	3.25	0.04	0.0013<br>(1 in 75,000 B cells)	998
Naïve Mouse #2	3.11	0.07	0.0021<br>(1 in 50,000 B cells)	1567

*Assuming 75 million B cells per mouse

Table S2: Kinetic parameters of ai-mAb interactions with iGL-VRC01 Fab as measured by BLI. Related to Figure 2.

Ligand	Analyte	K_D (M)	K_{on} (1/Ms)	K_{on} error (1/Ms)	K_{off} (1/s)	K_{off} error (1/s)
iv9*	iGL-VRC01 Fab	6.05E-09	2.24E+05	3.61E+03	1.35E-03	6.89E-06
iv9mod	iGL-VRC01 Fab	5.45E-06	2.74E+04	2.31E+03	1.46E-01	1.74E-03

*Data reproduced from Seydoux et. al.[34]

Table S3: Summary of mice and immunogens. N/A: not applicable. Related to Figures 1, 3, 4, & 5.

Related Figure	Mouse ID	Immunogen
Fig. 1	#1	N/A
	#2	N/A
	#3	N/A
Fig. 3, 4, and 5	#4	N/A
	#5	N/A
	#6	N/A
Fig. 3	#7	iv4/iv9
	#8	iv4/iv9
Fig. 3 and 4	#9	iv4/iv9mod
	#10	iv4/iv9mod
	#11	iv4/iv9mod
Fig. 5	#12	iv4/iv9mod
	#13	iv4/iv9mod
	#14	iv4/iv9mod

Table S4: Putative VRC01 precursors identified through cell sorting and sequencing of B cells from naïve and iv4/iv9mod-immunized ATX-GK mice. Related to Figures 1, 3, 4, and 6.

Cell ID	Tissue	Method and source	Gamma V-Gene	Gamma J-Gene	Gamma D-Gene	CDRH3	Kappa V-Gene	Kappa J-Gene	CDRL3
PATXP1	Spleen	High-throughput sequencing of naïve mouse #3	IGHV1-2*02	IGHJ4*02	IGHD6-6*01	CARGGYRQLVPFDYW	IGK3-20*01	IGKJ4*01	CQQYGTF
PATXP2	Spleen	High-throughput IgG+ sequencing of iv4/iv9mod-immunized mice #9 & #10	IGHV1-2*02	IGHJ6*03	IGHD3-22*01	CARDYYDSSGYYSLDYYYYMDVW	IGKV3-15*01	IGKJ4*01	CQQYNTF
PATXP3	Spleen	High-throughput IgG+ sequencing of iv4/iv9mod-immunized mice #9 & #10	IGHV1-2*02	IGHJ6*03	IGHD1-1*01	CARAQYNWNDGSYYYYYYMDVW	IGKV1-5*03	IGKJ1*01	CQQYNTF
PATXP4	Lymph nodes	Antigen-specific IgG+ sequencing of iv4/iv9mod-immunized mice #9, #10, &#11	IGHV1-2*02	IGHJ6*03	IGHD1-1*01	CARARYNWHDGSYYYYYHMDVW	IGKV1-5*03 or IGKV1-5*04	IGKJ1*01	CQQYNTF
PATXP5	Lymph nodes	Antigen-specific IgG+ sequencing of iv4/iv9mod-immunized mice #9, #10, &#11	IGHV1-2*02 or IGHV1-2*07	IGHJ6*03	IGHD1-1*01	CARAHYNWNDGSYFYYYYMDVW	IGKV1-5*03	IGKJ1*01	CQQYNTF

Table S5: Summary of all sorts of B cells from unimmunized and ai-mAb-immunized ATX-GK mice. Related to Figures 1, 3, 4, and 5.

Related Figure	Sorting Strategy	Sample	Tissue	Number of initial cells (M)	Number of B cells	IgG Frequency of Total B cells	Antigen-specific Frequency of IgG cells	Number of sorted cells	Number of sequenced HCs	Number of VH1-2 HCs	Number of sequenced LCs	Number of 5aa CDRL3 LCs	Number of paired sequences	Number of VRC01 precursors
Fig. 1	Unbiased	Naïve mouse #1	Spleen	N/D	16,500	N/D	N/D	N/D	4824	157	4832	2	4819	0
	Unbiased	Naive mouse #2	Spleen	N/D	16,500	N/D	N/D	N/D	5937	185	5924	4	5916	0
	iv4/iv9+ bulk sorted	Naïve mouse #3	Spleen	16	1,200,000	N/D	N/D	12146	1506	1440	1502	1	1502	1
Fig. 3	Bulk sorted IgG+ cells	Unimmunized mouse #4	Spleen	33	2,000,000	0.44	N/D	10518	27	1	33	0	27	0
		iv4/iv9-immunized mouse #7 and #8	Spleen	40	3,500,000	0.45	N/D	17403	2898	214	2950	3	2896	0
		iv4/iv9mod-immunized mouse #9 and #10	Spleen	20	2,100,000	0.54	N/A	8216	1684	81	1828	4	1677	2
Fig. 4 & 5	iv4/iv9mod+ IgG+ single cell sorted	Unimmunized mouse #4	Lymph node	10	100417	0.54	4.04	8	4	3	4	0	3	0
		Unimmunized mouse #5	Lymph node	5	17847	0.41	2.74	2	1	1	1	0	0	0
		Unimmunized mouse #6	Spleen	20	305609	0.41	2.91	13	10	10	8	0	8	0
			Lymph node	10	24733	0.59	1.39	0	0	0	0	0	0	0
Fig. 4	iv4/iv9mod+ IgG+ single cell sorted	iv4/iv9mod-immunized mouse #9	Spleen	10	240578	0.43	1.16	7	7	7	3	0	3	0
		iv4/iv9mod-immunized mouse #10	Spleen	10	818000	0.62	3.91	82	65	62	51	0	43	0
		iv4/iv9mod-immunized pooled #9, #10, and #11	Lymph node	10	179455	1.40	8.37	78	60	53	54	5	47	2
Fig. 5	iv4/iv9mod+ IgG+ single cell sorted	iv4/iv9mod-immunized mouse #12	Spleen	20	567000	0.39	3.27	21	20	20	18	0	17	0
		iv4/iv9mod-immunized mouse #13	Spleen	20	870000	0.38	4.73	53	43	43	42	0	33	0
			Lymph node	5	116238	0.72	3.71	11	0	0	0	0	0	0
		iv4/iv9mod-immunized mouse #14	Spleen	25	675334	0.42	2.52	25	24	23	22	0	13	0
			Lymph node	16	80505	0.49	4.50	5	4	3	2	0	1	0
